# Influence of Torrefaction on the Conversion Efficiency of the Gasification Process of Sugarcane Bagasse

**DOI:** 10.3390/bioengineering4010022

**Published:** 2017-03-10

**Authors:** Anthony Anukam, Sampson Mamphweli, Omobola Okoh, Prashant Reddy

**Affiliations:** 1 Fort Hare Institute of Technology, University of Fort Hare, Alice 5700, Private bag X1314, South Africa; smamphweli@ufh.ac.za; 2 Department of Chemistry, University of Fort Hare, Alice 5700, Private bag X1314, South Africa; ookoh@ufh.ac.za; 3 Department of Chemistry, Durban University of Technology, P.O. Box 1334, Durban 4000, South Africa; reddyp2@gmail.com

**Keywords:** sugarcane bagasse, torrefaction, gasification, efficiency, computer simulation

## Abstract

Sugarcane bagasse was torrefied to improve its quality in terms of properties prior to gasification. Torrefaction was undertaken at 300 °C in an inert atmosphere of N_2_ at 10 °C·min^−1^ heating rate. A residence time of 5 min allowed for rapid reaction of the material during torrefaction. Torrefied and untorrefied bagasse were characterized to compare their suitability as feedstocks for gasification. The results showed that torrefied bagasse had lower O–C and H–C atomic ratios of about 0.5 and 0.84 as compared to that of untorrefied bagasse with 0.82 and 1.55, respectively. A calorific value of about 20.29 MJ·kg^−1^ was also measured for torrefied bagasse, which is around 13% higher than that for untorrefied bagasse with a value of ca. 17.9 MJ·kg^−1^. This confirms the former as a much more suitable feedstock for gasification than the latter since efficiency of gasification is a function of feedstock calorific value. SEM results also revealed a fibrous structure and pith in the micrographs of both torrefied and untorrefied bagasse, indicating the carbonaceous nature of both materials, with torrefied bagasse exhibiting a more permeable structure with larger surface area, which are among the features that favour gasification. The gasification process of torrefied bagasse relied on computer simulation to establish the impact of torrefaction on gasification efficiency. Optimum efficiency was achieved with torrefied bagasse because of its slightly modified properties. Conversion efficiency of the gasification process of torrefied bagasse increased from 50% to approximately 60% after computer simulation, whereas that of untorrefied bagasse remained constant at 50%, even as the gasification time increased.

## 1. Introduction

The growth in world population and the economic development anticipated in developing countries such as South Africa are expected to contribute greatly to the accumulation of greenhouse gases (GHGs) in the atmosphere and its consequences with a direct influence on energy rivalry. Coal is the main source of energy in South Africa as approximately (ca.) 93% of the country’s energy needs are directly derived from coal, while about 92% is consumed on the African continent, with production mostly emanating from South Africa [1,2]. The utilization of coal deserves special attention considering the fact that its combustion alone accounts for about 30%–40% of atmospheric GHG emissions [3]. The interest in the development or modification of alternative sources of energy that rely on biomass materials such as sugarcane bagasse as feedstocks have been fuelled by the need to mitigate the negative environmental impacts linked to the use of coal. However, there are various transformation routes for biomass conversion into useable energy and other chemical products with paramount importance attached to the conversion efficiency of each of the different processes [4,5,6,7]. Nonetheless, several challenges are encountered when using biomass as an energy resource. These issues arise mainly as a consequence of the heterogeneity of the biomass in relation to their physical characteristics and chemical composition, which are also affected by the biomass origin. Therefore, to enhance the utilization effectiveness and efficiency of the conversion processes for biomass and the yield of selected products, pre-processing prior to conversion is necessary but the pre-processing method to be applied depends on the biomass conversion route [8,9,10,11,12]. For thermochemical conversion of biomass, torrefaction is considered an effective pre-processing method because it relies on heat-related treatment of the biomass at relatively mild temperatures (200–300 °C) in an inert atmosphere to increase the volumetric energy density, grindability and hydrophobic properties of the biomass, which can enhance the biomass conversion efficiency [13,14,15,16,17].

In comparison to coal, native biomass has lower elemental carbon and fixed carbon (FC) as well as lower higher heating value (HHV). Biomass has a HHV of ca. 15.20 MJ·kg^−1^, while that of coal is about 23.35 MJ·kg^−1^ [18,19]. When upgraded via torrefaction, biomass becomes a better quality solid fuel suitable for conversion into energy through gasification because the main constituents (cellulose, hemicellulose and lignin) are thermally degraded. The latter results in a material with better fuel quality since it leads to reduced content of volatile matter (VMC) from ca. 75.50 wt % to ca. 34.85 wt % or less, depending on the concentration of organic species in the biomass; while FC increases from ca. 10.74 wt % to ca. 13.45 wt % [20,21,22,23,24]. The concentration of the major elemental components (carbon and oxygen) of the biomass are also changed from about 41.52 wt % to above 45.68 wt %, and from around 44.63 wt % to about 11.45 wt % for carbon and oxygen respectively [23,25,26,27]. The wt % of torrefied biomass is usually in the range of 24–95 wt %; with a HHV between 16 and 29 MJ·kg^−1^ [18]. As a result, the yield of energy would be around 29.98% depending on temperature and time of torrefaction [18,28,29,30,31]. The gasification process of untorrefied form of biomass is lower in efficiency than that of its torrefied form, but additional cost may be incurred due to the heat and N_2_ required for torrefaction [32]. Therefore, to successfully commercialize torrefaction, a reduction in operating costs is vital.

Sugarcane is cultivated in many countries around the world, including South Africa, and the byproducts obtained during sugarcane processing have potential for value addition. In particular, a surplus amount of sugarcane bagasse (SCB) is generated by the sugar industries in South Africa (about 3.3 million tonnes/year), with most of it burned inefficiently in low-efficiency boilers as a way to get rid of the excess bagasse generated [33]. Numerous studies have been conducted on torrefaction of biomass for different purposes, but none has been geared towards the torrefaction of SCB for the purpose of gasification using the downdraft system. Chang et al. [34] investigated SCB subjected to torrefaction in an auger reactor at temperatures of 260, 280 and 300 °C, respectively. They reported that torrefaction results in the removal of moisture and some lightweight organic compounds from bagasse, indicating that temperature of torrefaction and the chemical composition of SCB had significant impact on product distribution. The impact of torrefaction temperature and residence time on the properties of different agricultural residues, which included SCB, was also investigated by Pimchuai et al. [35], who found that temperature had stronger impact than residence time in raising the energy density of torrefied SCB. A similar inference was also drawn by Bridgeman et al. [21], who studied the combustion behaviour of different biomass materials. The impact of thermal processing of pinewood at 260 °C in an inert atmosphere at varying residence times was also examined by Bourgois and Guyonnet [36], who concluded that the gases generated during the process of torrefaction were non-condensable gases that had CO, CO_2_, O_2_ and N_2_ as the main constituents from the results of chromatographic analyses. The weight of the torrefied material was also examined together with proximate and ultimate analyses and the findings showed that the mass of H and O decreased with variation in residence time between 15 min and 4 h. They also reported that the concentration of elemental C in the torrefied material increased with increasing residence time. Pentananunt et al. [37] also assessed the features of torrefied biomass in terms of proximate and ultimate analyses, including the density of the material. In their experiments, temperature and residence time varied from 250–270 °C and 2–3 h, respectively. They found similar results to those obtained in the study by Bourgois and Guyonnet, 1988 [36] in terms of H and O reduction as well as in terms of increased C content when torrefaction process temperature and residence time increased. They conducted a combustion test on the torrefied materials and found that torrefied biomass showed more appropriate behaviour because it produced less dense smoke, less soot and higher speeds during combustion when compared to raw biomass.

However, in comparison to combustion, it is widely recognized that gasification technology is more efficient and environmentally friendly in terms of operation and is considered to be an important route for the conversion of SCB into energy. The gasification of lower-quality fuels results in poor gas quality and a high concentration of tar [38]. Pre-processing of biomass, employing torrefaction, is necessary to address the problem of low quality of biomass for efficient energy conversion. Torrefied biomass can be successfully pulverized to allow for physical transformation into a suitable condition for gasification or even co-gasification with coal, if the need arises [16,39]. A study by Prins et al. [40] reported that torrefied wood can be more efficiently gasified than raw wood in an oxygen-blown entrained flow gasification system. Gas yield and reaction kinetics were examined by Couhert et al. [41] during gasification of torrefied beech wood using steam as the gasifying agent in an entrained flow gasification system. They reported that torrefied wood produced more H_2_ and CO than the raw feedstock. This research work therefore seeks to study the influence of torrefaction not just on the features of SCB but also on the conversion efficiency of its gasification process under standard conditions of gasification with a view to compare and establish the feedstock (torrefied or untorrefied SCB) that would be more suitable for gasification based on efficiency. The gasification process of both torrefied and untorrefied SCB relied on computer simulation in which a software programme specifically designed for downdraft gasification systems was used.

## 2. Materials and Methods

### 2.1. Sample Preparation

The sugarcane bagasse (SCB) used for this study was obtained from a local sugarcane mill in KwaZulu-Natal province of South Africa, with ca. 40 wt % moisture content as received. The SCB was air-dried outdoors at ambient temperature of about 32 °C for seven days to lower its moisture content. The dried SCB was ground using a cryogenic grinder to a small particle size range of 1–2 mm and subsequently sieved to obtain a mean size of 20 to 100 μm in order to maintain uniformity as required by the instruments used for analysis. Besides, irregular biomass size results in non-uniform torrefaction that may create heat and mass transfer delays [42,43]. The sample was placed in an airtight vial and stored in a desiccator for further analyses. According to Ostermeijer [44], the quantities of torrefied biomass that are used in demonstration and lab-scale level are inadequate to test torrefaction products at a commercial scale.

It is important to note that sugarcane bagasse (SCB) and bagasse were used interchangeably throughout this paper. These apparently mean the same thing.

### 2.2. Torrefaction Process

**Figure 1:**
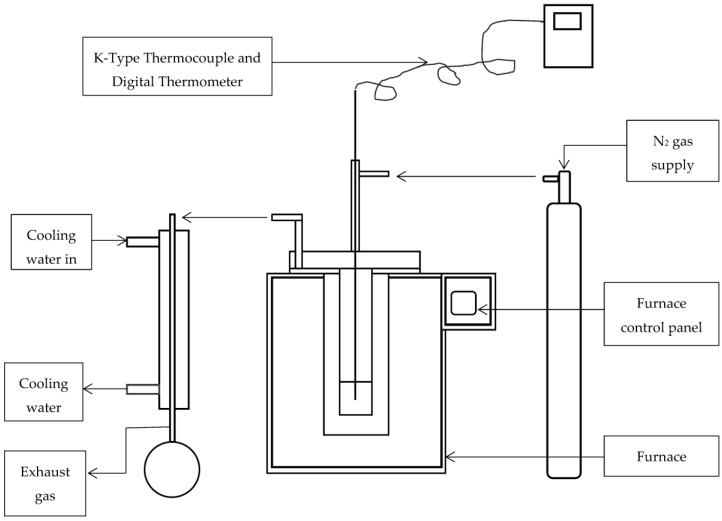
A schematic diagrammatic representation of the equipment used for torrefaction of bagasse. Reproduced with permission from [45].

Torrefaction of SCB was conducted in a laboratory scale batch torrefaction muffle furnace connected to a system that supplies N_2_ gas. A maximum of 0.5 kg of sample can be torrefied at a given time using this furnace. The furnace is ca. 42 cm in diameter, about 66 cm in height and 49 cm in length. Its combustion chamber is around 26 cm in diameter and 20 cm in height and length. The experimental setup consisted of a tube-type vessel made of stainless steel that is designed to fit inside of the furnace, a sample holder that also fits inside of the tubular vessel as well as a condenser for the collection of released gases. The maximum working temperature of the furnace is about 3000 °C. With the sample inside the furnace, the experiment was performed at 300 °C for 1 h. A simplified diagrammatic representation of the furnace used for torrefaction is presented in Figure 1.

**Figure 2:**
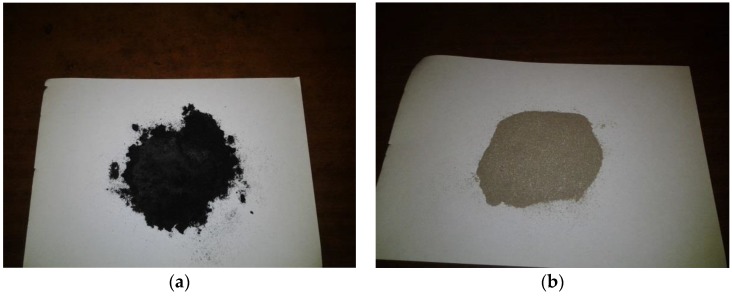
Images of sugarcane bagasse: (**a**) Torrefied at 300 °C with at a residence time of 5 min; (**b**) untorrefied.

About 15 g of SCB was weighed and placed on a sample holder and mounted to the inner cylinder of the tube-type vessel. A connection between the vessel and the inlet N_2_ gas supply system as well as a condenser was established. N_2_ gas was blown via the tube-type vessel at a flow rate of 5 L·min^−1^ to purge the system of any oxygen in the system and eliminate the presence of volatiles in the tube. The N_2_ gas flow rate was then reduced to about 0.5 L·min^−1^ to prevent the volatiles and gases from completely escaping into the atmosphere as some of these were collected for analyses after the experiment; however, the main function of the N_2_ supplied to the torrefaction process, since it condenses and freezes at a particular temperature, was to prevent the furnace from overheating. A preheating of the furnace to a set temperature was undertaken before the tubular reactor containing the sample was placed inside the furnace for the torrefaction process to take place. As the torrefaction experiment progressed in the furnace, the sample heating rate in the furnace was pre-set to 10 °C·min^−1^, which is characteristic of gasification process heating rate linked to fixed bed gasifiers. The experiment was conducted at a maximum temperature of 300 °C for an hour, which was counted from the time the experiment commenced at room temperature to the time the sample temperature inside the furnace attained 300 °C. Sample residence time of 5 min was maintained in the vessel to allow for rapid reaction and cooling of the sample while still in the furnace, which eliminates the possibility of partial combustion of the sample during its cooling under air atmosphere. This residence time was made relatively short to prevent severe decomposition of the sample while still in the vessel. After the torrefaction experiment and as the residence time elapsed, the entire vessel was withdrawn from the furnace and allowed to cool in the ambient air. The solid product (sample), referred to as the torrefied material was also removed from the vessel chamber, cooled and weighed to determine the solid yield. The solid sample was placed in airtight vials and preserved in a desiccator for further analyses. The volatiles produced in the process were collected and cooled in the condenser, while the non-condensable gases were collected at 15-min intervals in small gas sampling bags for analysis. Figure 2a,b show the torrefied and untorrefied bagasse.

The mass and energy yield of the torrefied bagasse were also determined. These were calculated on a dry and ash-free basis (daf) according to Equations (1) and (2) [46]:

(1) $$Mass\;yield\;\left(wt\;\%\right)=\left(\frac{M_{torrefied}}{M_{untorrefied}}\right)\times 100\%$$

(2) $$Energy\;yield\;\left(wt\;\%\right)=Mass\;yield\times \left(\frac{CV_{torrefied}}{CV_{untorrefied}}\right)\times 100\%$$

where *M* is the mass and *CV* is the calorific value of torrefied and untorrefied bagasse, respectively.

### 2.3. Product Characterization

All tests conducted in this study were repeated at least three times and the results presented are represented by the average of these tests, and where applicable, the standard deviation for the properties measured is also presented.

#### 2.3.1. Analysis of Gaseous Products

An investigation of the gaseous products from torrefaction of bagasse was undertaken in order to determine the composition and yield of the products at a torrefaction reaction temperature of 300 °C. This is necessary to compare the composition of the gaseous products generated during torrefaction of SCB with those produced during its gasification.

The non-condensable gases produced from torrefaction of bagasse were analysed by a Perkin Elmer Autosystem XL gas chromatographic (GC) instrument (Perkin Elmer, New York, NY, USA). Argon was used as a carrier gas at a flow rate of 35 mL·min^−1^, with gas composition analysis carried out using mixed standard gas as an external standard.

#### 2.3.2. Solid Product Analysis

The yield of solids after torrefaction of biomass is a measure of the biomass resistance to thermal decomposition, which is defined according to Equation (1) [47]. The torrefied sample was weighed and a value of 12.3 g obtained.

The yield of the solid product was obtained using Equation (1). The sample was then analysed in terms of proximate and ultimate analyses as well as in terms of calorific value to quantify the amount of energy available for conversion. Results of these analyses were used to conduct computer simulation of the gasification process of torrefied SCB and the gasification process results compared with those of untorrefied SCB.

#### 2.3.3. Proximate and Ultimate Analysis

Biomass feedstock properties relevant to gasification are usually described in terms of proximate analysis, which separates the biomass into four categories (moisture, volatile matter, fixed carbon and ash) that are of importance to thermal conversion of biomass materials; and ultimate analysis which provides the relative copious quantity of individual elements such as C, H, O, N and S that are contained in the material [48].

The proximate analysis parameters were determined from thermogravimetric analysis plots following a modified method of the ASTM D 5142-04 standard test method [49,50]. Moisture content was determined by weight loss at temperatures lower than 100 °C, while the content of volatile matter represented the mass evolved between the temperatures of 100–1000 °C. After oxidative heating of the sample to about 1000 °C during TG analysis, the remaining mass was considered ash. The FC amount was obtained by difference.

The concentration of C, H, N and S in the samples was measured in a ThermoQuest CHNS elemental analyser (ThermoQuest, Manasquan, NJ, USA). About 10 mg of torrefied bagasse was mixed with an oxidizer in a tin capsule which was combusted afterwards in a reactor at around 1000 °C. A violent reaction in a temporary enriched O_2_ environment was promoted as the sample and container melted. Tungsten trioxide (WO_3_) and copper (Cu) were used as catalysts downstream of the combustion chamber to ensure complete oxidation. The catalysts’ oxidizer (WO_3_) and reducer (Cu) were both kept at a temperature of 1000 °C. Combustion products such as CO_2_, SO_2_ and NO_2_ were produced in the process and were conveyed by a constant flow of helium-rich carrier gas. At the set temperature, NO_2_ was catalytically reduced to N_2_, while the other components (C, H and S) were left in their oxidized forms, which were CO_2_, H_2_O and SO_2_, respectively. These gases were then separated by GC and measured with a thermal conductivity detector.

#### 2.3.4. Calorific Value Determination

Calorific value, also known as higher heating value, is a feedstock property that affects the effectiveness of thermochemical conversion systems and remains a primary measure of the quality of biomass as a fuel for energy production; it is the energy accessible in the biomass as an estimate of the heat liberated during full combustion of the biomass to CO_2_ and H_2_O [48].

The CV of torrefied and untorrefied bagasse was calculated using an equation developed by Sheng and Azevedo, 2005 [51]. This is represented by Equation (3):

(3) $$CV\;\left(MJ\cdot kg^{-1}\right)=-1.3675+0.3137\times C+0.7009\times H+0.0318\times O$$

where *CV* is the biomass calorific value in MJ·kg^−1^; *C* represents the weight percentage (wt %) of carbon in the biomass as determined by ultimate analysis; *H* depicts the weight percentage of hydrogen in the biomass as determined also by ultimate analysis; *O* is the weight percentage of oxygen determined by difference from ultimate analysis on a dry and ash free basis, i.e., O=100−(C+H+N). Because of a lack of specialized analytical instruments for the determination of oxygen, its weight percentage is usually determined by difference in terms of the weight fractions of other elemental components.

#### 2.3.5. Thermogravimetric Analysis

The temperature at which reactions leading to decomposition of the samples begin can be monitored by a thermogravimetric analyser (TG). The weight loss (TG) and its derivative plot (DTG) of the samples have been measured as a function of temperature. This analysis was undertaken not just to establish the thermal stability and gasification temperature of SCB, but also to determine the major parameters that influences thermal conversion of SCB.

A Perkin Elmer TGA 7 (Perkin Elmer, Norwalk, CA, USA) was used to study the thermal degradation behaviour of torrefied SCB. A 3.288 mg of the sample was dispersed flatly on a crucible with a flat bottom of ca. 8 mm in thickness and 3 mm in depth. The sample was heated over a wide temperature range of 20–1000 °C in the presence of a non-oxidizing atmosphere of N_2_ at 10 °C·min^−1^ heating rate. An inconsequential amount of sample and a low heating rate were used to avoid limitations in heat transfer and to minimize the impact of mass transfer. Another reason for the low heating rate used was because it is a characteristic of fixed-bed gasification systems.

It is imperative to note that the thermal conditions used in TG are different to those used during torrefaction because the former was used to study the thermal behaviour of the sample over a wide temperature range, since the purpose of TG analysis was to use the sample in a gasification process, which is also a high-temperature energy conversion process; the latter process (torrefaction) was used to improve sample characteristics under mild temperature conditions. The maximum temperature for this process (torrefaction) was 300 °C. However, the f reaction beginning for both torrefaction and TG was at room temperature (20 °C).

#### 2.3.6. Scanning Electron Microscopic Analysis

Morphological characterization of the samples were undertaken to determine the effect of torrefaction on the micro and macro-structural transformation of SCB. These were examined using a SEM model JEOL (JSM-6390LV) (JEOL, Tokyo, Japan). Samples were gold coated prior to analysis using a sputter coater (Eiko IB3 Ion coater, Eiko Engineerin Co. Ltd., Tokyo, Japan) and placed on a specimen holder called a stub by a carbon double-sided tape and mounted in the sample chamber of the instrument for morphological viewing. The SEM instrument requires that samples be coated with an ultra-thin coating of electrically conducting materials, commonly gold (Au), to give a good yield of secondary electrons, which will in turn result in a good-quality image. Another reason for Au coating is to minimize accumulation of surface charges that could reduce resolution. To reduce errors and confirm the results, each analysis was repeated in triplicate under the same conditions. Included in each one of the micrographic images of the torrefied and untorrefied bagasse are specific examination conditions of the analysis.

#### 2.3.7. Gasification Simulation

Experimental procedures involving actual gasification of SCB are quite expensive and time consuming hence the use of fast and quick simulation techniques for the gasification process of bagasse, which is much less expensive.

A biomass gasification simulation programme was used to conduct computer replicate of the gasification processes of torrefied and untorrefied SCB. The computer software programme was based on a model specifically designed for downdraft systems to evaluate the influence of operating and design variables on the operation of the gasifier. The software was developed by Chen et al. [52] and modified by Jayah et al. [53]. It consists of two submodels in the form of flaming pyrolysis and gasification zone submodels. The flaming pyrolysis zone submodel is often used to determine the product concentration and temperature of the gas leaving the flaming pyrolysis zone, while the gasification zone submodel is used to predict the output of the product gas and the length of the gasification zone at any given time [53]. A detailed description of the gasification simulation programme was presented in a previous paper [38]. Gas profiles obtained after simulation were used to compute the heating value of the syngas from the percentage composition of the combustible gases contained in the syngas as follows in Equation (4) [54]:

(4) $$CV_{gas}=\left[\frac{\left(CO_{vol}\times CVCO\right)+\left(H_{2vol}\times CVH_{2}\right)+\left(CH_{4vol}\times CVCH_{4}\right)}{100}\right]$$

where *CV_gas_* is the gas calorific value in MJ·Nm^−3^, *CO_vol_* is the volume concentration (in %) of carbon monoxide gas, CVCO is the calorific value of carbon monoxide gas (usually 12.64 MJ·Nm^−3^ by standard) [55], *H_2vol_* is the volume concentration (in %) of hydrogen gas, *CVH_2_* is the calorific value of hydrogen gas (10.1 MJ·Nm^−3^ by standard) [56], while *CH_4vol_* is the volume concentration (in %) of methane gas, *CVCH_4_* is the calorific value of the methane gas (usually 38 MJ·Nm^−3^ by standard) [55]. The calorific values of the combustible gases were obtained from the standard gas table.

The conversion efficiency of the gasifier was determined after computer simulation of the gasification process by Equation (5) [54]:

(5) $$\eta =\left[\left(\frac{CV_{gas}\times 2}{CV_{fuel}}\right)\times 100\%\right]$$

where *η* is the efficiency (in %) of the gasifier, *CV_gas_* is the gas calorific value, and *CV_fuel_* is the calorific value of SCB. The factor ‘’2’’ in Equation (6) represents the gas flow rate from the gasifier and is measured in Nm^3^·h^−1^ [53]. The parameters used during gasification simulation of torrefied and untorrefied SCB are provided in Table 1.

The throat in downdraft gasifiers is a special unique feature of the gasifier responsible for even heat distribution around the combustion zone of the gasifier and consequently along the gasification axis. Therefore, throat angle and diameter in Table 1 basically refer to gasifier geometry in relation to the cross-sectional area at a certain height of the gasifier. Insulation thickness refers to resistance to conductive heat flow inside the gasifier, and thermal conductivity denotes the ability to conduct heat outside the gasifier. The temperature of the input air, measured in Kelvin (K) temperatures, and air input into the gasifier, measured in kg·h^−1^, are actually different parameters. While the former represents the temperature of the air blown into the gasifier, the latter depicts the quantity of air introduced into the gasifier in kilograms per hour. Note that gasifiers are generally operated at an ambient air temperature of 27 °C (300 K) [54]. Feed input is the quantity of SCB fed into the gasifier, while the value 12.8% is an indication of the amount of heat lost, which can be recovered through other methods; however, this is beyond the scope of this study, which requires further investigation to actually determine the methods that can be used for heat recovery.

It is also worthy to note that particle diameter for both torrefied and untorrefied SCB was assumed as the same since feedstocks needs to be briquetted prior to gasification. This briquette form is almost impossible to achieve under gasification processes that rely on computer simulation, hence the need to assume the particle diameter/size. On the other hand, it is also almost impossible to gasify fine particle feedstocks as doing this would create many technical issues including lack of gravity feeding within the gasifier, combustion instead of gasification and so on [54].

## 3. Results

The results from this study are presented in the following subsections. Information relating to temperature profiles was reported in a previous publication in which the impact of temperature on various parameters during torrefaction of SCB was investigated [57].

### 3.1. Product Distribution from Bagasse Torrefaction

**Figure 3:**
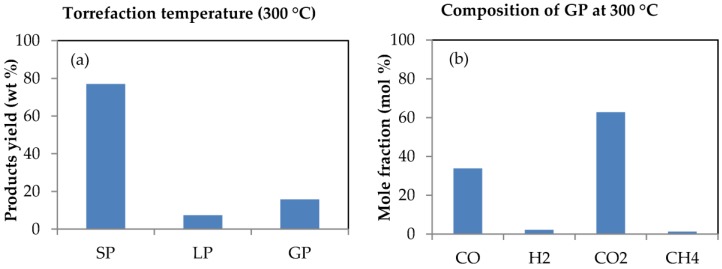
Products obtained from bagasse torrefaction: (**a**) Product yield with SP, LP and GP representing the solid, liquid and gaseous products, respectively; (**b**) composition of gaseous products formed from sugarcane bagasse torrefaction at 300 °C, and at 5 min time of residence.

Quite a number of reaction products are usually formed during torrefaction of biomass and their yield is heavily dependent upon the conditions of torrefaction including temperature, time and heating rate as well as the characteristics of the biomass [58]. The products from the torrefaction of SCB were identified as consisting of solid product (the torrefied bagasse), condensable liquid and gaseous products. The analysis was selected so that it would sufficiently describe the conditions existing in a gasification process. Figure 3a and b presents the impact of torrefaction on the yield of products as well as on the composition and mole fractions of the gaseous products formed from sugarcane bagasse (SCB) torrefaction.

Torrefaction of biomass above 300 °C results in extensive devolatilisation and initiates pyrolysis [59]. This was why, as previously mentioned, a torrefaction temperature not exceeding 300 °C was chosen for this study. The impact of temperature on various torrefaction parameters and their relevance to gasification had been investigated in a previous publication [60]. However, as is evident in Figure 3a, almost 80% of torrefied SCB was retained as solid product compared to the lower yields of condensable liquid and gaseous products with percentage yields of 7.3% and 15.7%, respectively, which agrees with the observation that about 70%–80% of the biomass is usually retained after torrefaction, as reported by Luo [45]. The mass of torrefied SCB retained as solid product was due to the fact that torrefaction was conducted in a non-oxidative environment; this mass implies about a 20% reduction in weight, and was ascribed to changes occurring in the structure of hemicellulose during torrefaction of the sample. The loss in weight of biomass during torrefaction significantly depends on temperature as a consequence of the breakdown of the cellulose and hemicellulose content of the biomass; and the thermal degradation temperature of these components fall within the temperature range of 150–500 °C [15,61,62]. This temperature range also accommodates the temperature employed for the torrefaction process of SCB (300 °C). Considering gasification as a high temperature process, this sort of thermal behaviour for SCB during torrefaction is also expected during its gasification, however, the extent of the yield of products would be largely dependent upon the conditions of gasification as well as upon the source of the SCB, which would ultimately influence its composition and characteristics [53,63].

The effect of torrefaction is also evident on the composition of gaseous products formed from SCB torrefaction. This is obvious from Figure 3b as the gaseous products mainly contain CO_2_, CO and traces of H_2_ and CH_4_ in varying proportions due primarily to the thermal degradation of hemicellulose together with some short-chain lignin components that are among the main chemical constituents of biomass materials [58]. Torrefaction becomes entirely exothermic at temperatures above 280 °C, leading to the production of varying proportions of gases such as CO, H_2_, CO_2_ and CH_4_ [59]. This is in agreement with the varying fractions of gases obtained from this study during torrefaction of SCB. At a torrefaction reaction temperature of about 300 °C, CO_2_ formation was mainly due to decarboxylation reactions as a result of unstable carbonyl groups in the hemicellulose structure of the torrefied material, while secondary reactions of CO_2_ and steam with permeable char together with low molecular weight carbonyl compounds produced from decarbonylation as a result of torrefaction explains the formation of CO [64,65,66]. However, the CO was more aggressively formed owing to the aforementioned secondary reactions. The low H_2_ composition was attributed to the low moisture content of torrefied SCB. H_2_ production increased with increasing material moisture content and temperature (since the torrefaction process was monitored from room temperature up to 300 °C) as a consequence of rising endothermic reactions during torrefaction [67,68,69]. The production of CH_4_ was also mainly as a result of the formation and consumption of CH_4_, which both took place at high temperatures because of reduction in the composition and formation of CH_4_ as temperature rose; CO, H_2_, CH_4_, and CO_2_ are the major constituents of the syngas produced during gasification of biomass and their composition and yield is dependent on several process factors, the most important being temperature [70].

### 3.2. Mass and Energy Yield

**Figure 4:**
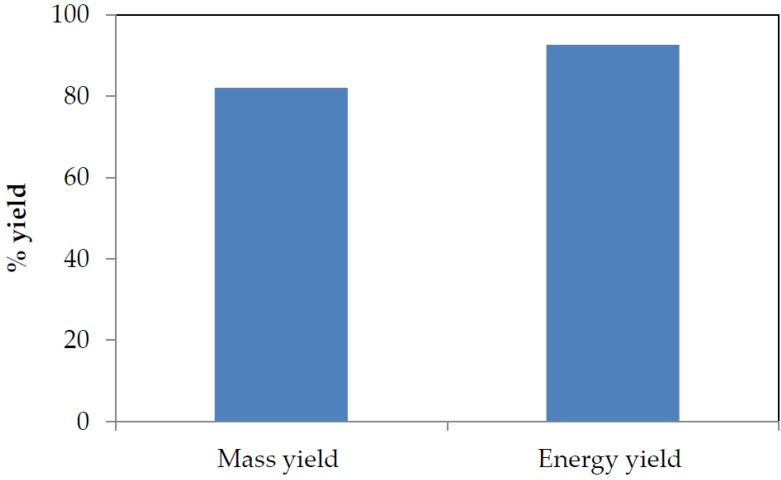
Mass and energy yield from SCB torrefied at 300 °C at a residence time of 5 min.

Considering torrefaction as one method to improve biomass properties for energy production purposes, input and output parameters must be carefully balanced. These input and output parameters are often measured in terms of mass and energy yield, which are main parameters used to evaluate torrefaction processes because they serve as medium used to describe the transition from mass and chemical energy from the biomass to the solid product [58]. The intensity of torrefaction of SCB was evaluated in terms of mass and energy yield. These parameters are also of utmost importance for the design and final optimization analysis of gasification plants involving the value chain between raw and torrefied biomass [71]. Figure 4 shows the impact of torrefaction on the mass and energy yields from SCB torrefaction process. These mass and energy yields were obtained from Equations (1) and (2), respectively.

In Figure 4, high mass and energy yields in the solid product can be observed, which was due to the release of volatiles and the residence time allowed for the torrefaction process (5 min) as well as the performance of the torrefaction experiment in a non-oxidative environment. Non-oxidative torrefaction always gives higher mass and energy yields than oxidative torrefaction irrespective of the type of biomass torrefied [47]. These parameters (mass and energy yield) are a consequence of the destruction of SCB fibrous structure that was attributed to decomposition reactions, which renders the sample dried and energy-dense. Torrefaction leads to an increased mass yield with improved energy properties of biomass; based on a dry and ash-free basis, the yields can be up to 80% for mass yield, and ca. 90% for energy yield [72,73,74]. The high mass and energy yields obtained imply that torrefied SCB would favour high temperature gasification that would result in optimum gasification efficiency as feedstock conversion into the desired products is described by the mass and energy yields of the gasification process [75].

### 3.3. Physical, Chemical and Calorific Value Analyses

As previously mentioned, proximate analysis parameters are important indices of solid fuels intended for energy production purposes. The key properties relating to efficient thermal conversion of torrefied and untorrefied SCB are presented in Table 2. The standard deviation values for the measured properties are also presented, which were determined from the mean of the measured properties and taking the positive square root of their variance.

It is evident from Table 2 that the two materials (torrefied and untorrefied bagasse) exhibit different properties, especially in terms of volatile matter content and fixed carbon as well as in terms of C and O contents including calorific values. The relative proportions of the content of volatile matter and fixed carbon are linked to the yields and composition of solid, liquid and gaseous products formed during gasification [76]. Volatile matter content decreased from 71.73%, in the untorrefied SCB, to 30.07% in torrefied SCB, which concurs with the fact that torrefaction reduces the volatile matter content of biomass, as reported by Sarkar et al. and Li et al. [77,78,79]. Fixed carbon also increased in torrefied SCB compared to untorrefied SCB. The ash content also increased by 92% (from 1.32% to 16.61%) in the torrefied material and the observed increases in ash and fixed carbon contents were mainly attributed to a concentration effect due to mass loss, leading to an increase in the mineral matter content of torrefied SCB, which are major contributors to ash composition [48]. During the torrefaction of biomass, alkali and alkaline earth metals, which are mostly ash-forming elements, are usually retained in the torrefied material, mainly as a result of the relatively low devolatilisation temperature of the process [80,81]. The moisture contents of both samples are relatively low and attributed to drying before analysis, which further reduced the level of moisture in the samples. To render biomass materials suitable for conversion processes, the moisture content must be adjusted as uncontrolled variations may lead to reduced process efficiency and increased cost [82].

The samples also differ in terms of elemental composition as results show that untorrefied bagasse is characterized by high O content and relatively low C and H_2_ contents. Appreciable amounts of O and other elements make raw biomass thermally unstable, at the same time producing considerable quantities of tar that could create problems that may lead to lower heat and mass transfer rates during gasification [83,84]. On the basis of these weight fractions, untorrefied SCB can be classified as a low calorific value biomass material. The reduction in O and H contents of torrefied SCB is the reason for the increase in its content of C. This makes the torrefied material more suitable as fuel for energy production [85,86]. It is also evident from Table 2 that torrefied SCB has a slightly lower O–C ratio (0.50) compared to untorrefied bagasse (0.82). This slight difference could be linked to the content of cellulose and lignin in the materials. A higher ratio of lignin–cellulose in biomass reflects a reduced H–C ratio as well as a lower O–C ratio; specific H–C stoichiometry is required for upgrading gasification products [87,88]. However, the alteration in the chemical composition of torrefied SCB resulted in a slight decrease in its O–C ratio. Lower O–C ratios lead to improved gasification characteristics of torrefied biomass [40]. The H–C ratio of torrefied SCB is also quite lower, with a value of 0.84, than that of untorrefied SCB whose H–C ratio is ca. 1.6. In relation to gasification, this implies that full conversion of the torrefied material would require the addition of supplemental materials such as H_2_ in the form of steam, or removing C in the form of CO_2_ [89,90].

The calorific value results are also presented in Table 2, showing the influence of elemental composition on the calorific value of torrefied SCB. The calorific value increased by approximately 12% in torrefied SCB when compared with the calorific value of untorrefied SCB. This was attributed to the reduced O–C and H–C ratios as well as the high content of C. Low O–C and H–C ratios increase the calorific value of biomass materials after torrefaction and make it akin to that of coal [91]. That of untorrefied SCB is much lower, probably due to its high content of oxygen. Oxygen contributes little or no energy during thermochemical conversion of biomass and decomposes following thermal treatment; optimum conversion efficiency of a gasification process is achieved with feedstock having high calorific value [51,38,92].

### 3.4. Thermal Characteristics

**Figure 5:**
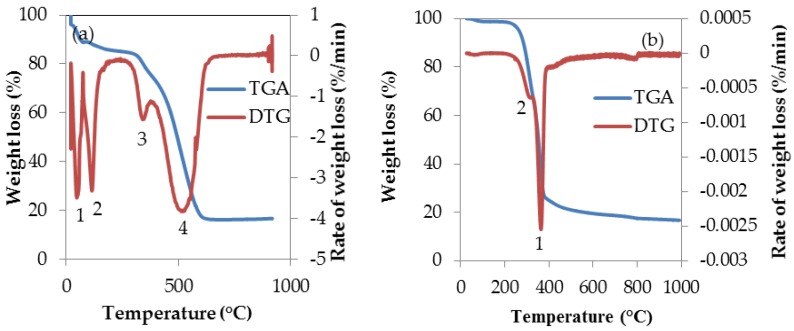
TGA and DTG plots of SCB obtained at 10 °C·min^−1^ heating rate: (**a**) torrefied SCB; (**b**) untorrefied SCB.

The thermal stability and combustion behaviour of torrefied and untorrefied SCB were investigated by TGA and its derivative (DTG). The weight loss and the rate at which it occurs are presented as a function of temperature. These are both presented in Figure 5a,b.

The three major constituents of biomass are cellulose, hemicellulose and lignin. Due to the structural differences of these constituents, they are commonly distinguished and identified by the use of TGA and the temperature ranges for the decomposition of these constituents had been measured in previous studies by other researchers [93,94]. The decomposition of hemicellulose occurs at 220–315 °C, and cellulose at 315–400 °C, while lignin decomposes over a wide temperature range beginning from 160–900 °C [95].

The TGA and DTG curve of torrefied SCB sample show a complex thermal degradation process involving several steps (1–4) that began at ambient temperature and ending at a temperature close to 1000 °C, while that of untorrefied SCB show two distinct weight loss stages (numbered 1 and 2) as depicted by the DTG peak. However, the rates of weight (R_W_) loss described by the DTG for both torrefied and untorrefied SCB shows that R_W_ is ˂0 in Figure 5a (for torrefied SCB), and ˃0 in Figure 5b (for untorrefied SCB). The reason for this difference in R_W_ is probably attributed to the nature of the samples as the characteristics of torrefied SCB was altered due to torrefaction compared to the characteristics of untorrefied SCB, which may have impacted on the thermal decomposition process of torrefied SCB.

The first step of weight loss began at ca. 25 °C and 98 °C for torrefied and untorrefied SCB respectively. The difference in the initial weight loss at the said temperatures was due to thermal pre-treatment of SCB prior to TGA analysis as a consequence of discrepancies in the chemical composition of both samples. This initial weight loss stages can be attributed to moisture evaporation from the samples [38,96]. The second, third and fourth weight loss stages for torrefied SCB are a reflection of the thermal decomposition of other components as well as carbonization [97]. The remaining final product, which is thermally stable at approximately (ca.) 1000 °C, was considered as ash. As previously stated, from Figure 5b, it can be clearly observed that the thermal degradation process of untorrefied SCB is quite different from that of torrefied SCB. The first weight loss corresponding to this curve was previously explained; however, the second peak indicates the beginning of volatile release around 260 °C. From the DTG of torrefied SCB (Figure 5a), maximum weight loss occurred at ca. 550 °C, as depicted by the broad peak (stage 4 weight loss). This was as a result of thermal decomposition attributed to the release of volatiles and the decomposition of hydrocarbons and FC as well as char gasification since studies on biomass materials involving TGA are often considered the same as studies on char gasification [98]. For untorrefied SCB, maximum weight loss occurred at 360 °C, owing mainly to the loss of volatiles that was also due to the decomposition of the major chemical components of the sample such as cellulose, hemicellulose and lignin [34].

The thermal degradation process of torrefied SCB was attributed mainly to decomposition of fixed carbon, whereas in the case of untorrefied SCB, its thermal decomposition process was dominated by volatile matter decomposition and emission, due obviously to higher volatile matter content. The higher the content of volatile matter of biomass, the easier its ignition and decomposition [99]. In relation to gasification, however, the analysis successfully established the thermal parameters that would influence the gasification of both torrefied and untorrefied SCB. These parameters are temperature, time and heating rate. According to the plots in Figure 5a and b, the maximum decomposition temperature for torrefied SCB is ca. 860 °C, while for untorrefied SCB, maximum decomposition temperature is about 1000 °C, with torrefied SCB displaying multiple weight loss stages compared to untorrefied SCB, which was attributed to change in characteristics as a result of torrefaction. This implies that, during gasification at the aforementioned temperatures, both materials would have completely degraded, leaving a certain amount of byproducts (such as ash, tar and soot) whose composition and yield would be affected by the composition of both samples as well as by the conditions of gasification.

### 3.5. Morphological Characterization

**Figure 6:**
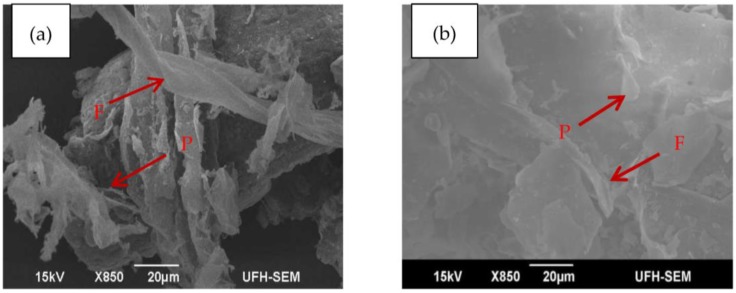
SEM images of SCB obtained under the same analysis condition with: (**a**) torrefied; and (**b**) untorrefied.

The micro and macrostructure of the samples were examined with the purpose of gaining a deeper insight into the effect of torrefaction on the structural transformation of the torrefied material and compare this transformation to the untorrefied material. The SEM images of the torrefied and untorrefied samples of SCB were taken under the same analysis condition (magnification was ×850 at 15 kV) for best comparison results. The images are presented in Figure 6.

From Figure 6, it is evident that the microscopic structures of the two samples are different. In terms of surface morphology, torrefied SCB appears darker in colour with rough surface compared to untorrefied SCB with lighter and smooth surface. However, no morphological changes are noticeable on the surface of the structure of untorrefied SCB (Figure 6b) due to lack of pre-treatment prior to analysis, whereas the reverse is the case for torrefied SCB (Figure 6a) as morphological changes are quite evident on the surface of its structure. This obviously can be attributed to thermal treatment. Nonetheless, two major morphological features are clearly shown in the images of both samples and are indicated by the arrows. These are fibre structure and pith, which are clearly denoted by the letters “F” and “P” in Figure 6a,b, respectively. The noticeable pith in Figure 6b is somewhat ruptured after thermal treatment, as indicated by the arrow in Figure 6a. Parallel stripes that are partially covered with residual material form the main part of the surface of the fibre in Figure 6a. The image of torrefied SCB also shows a highly disordered carbon structure that may promote high reactivity during gasification [100]. The fibrous structure (F) in Figure 6b is clearly exhibited in Figure 6a; this was due mainly to hemicellulose and lignin consumption during torrefaction. The variation in the macro- and microstructure explains why the grindability of torrefied biomass is superior to that of untorrefied biomass [15]. A more permeable structure with larger surface area could also be observed from the image of torrefied SCB compared to untorrefied SCB. These are favourable properties that lead to high conversion efficiency during gasification, and confirm that torrefied SCB is a carbon-based feedstock that is more suitable for gasification in a downdraft system appropriately developed to harbour the feedstock characteristics.

### 3.6. Gasification Simulation Process

**Figure 7:**
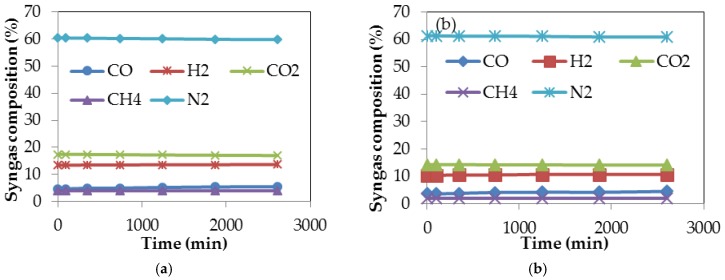
Syngas composition obtained after computer simulation of the gasification processes of (**a**) torrefied SCB; and (**b**) untorrefied SCB.

The conversion efficiency of a gasification process is an important factor that determines the actual technical operation as well as the economic viability of using a gasification system; it is defined as an expression of the energy content of the gaseous products formed during gasification, to the energy content of the biomass used as feedstock [54]. The software programme described in Section 2.3.7 was used to undertake computer simulation of the gasification processes of both torrefied and untorrefied SCB under standard gasifier operating conditions. The parameters used during gasification simulation are presented in Table 1. The simulation programme was used to establish the percentage composition of the syngas produced from the gasification processes of both torrefied and untorrefied SCB. Figure 7a,b shows the percentage composition of the syngas produced during gasification of torrefied and untorrefied SCB obtained after computer simulation.

It is quite evident from Figure 7a that the gases produced during gasification of torrefied SCB are, in terms of composition, comparable to those produced during its torrefaction (presented in Figure 3b). The same gases were also produced even for the gasification of untorrefied SCB (Figure 7b). These gases collectively represent the composition of the syngas; implying that feedstock pre-treatment does not change the end product of a gasification process. Syngas remains the end product of gasification irrespective of the type of pre-treatment method applied prior to gasification, but its concentration and quality may vary accordingly [25]. From Figure 7a,b, it is also clear that there are not many differences in the percentage composition of the constituents of the syngas obtained after gasification of torrefied and untorrefied SCB; however, the composition of the gases remained constant even as gasification time increased. N_2_ composition, approximately 60%, represents the gas with the highest composition due to syngas dilution, with N_2_-containing air used as the gasifying agent during computer simulation of the gasification processes of both torrefied and untorrefied SCB. The composition of the syngas produced during biomass gasification is a function of gasifier type and the gasifying agent used, as syngas produced using steam as the gasifying agent will have a lower percentage of N_2_ (around 3%) compared to that produced when air is used as the gasifying agent, which will usually have a N_2_ composition above 41%–50% [101]. There was a reduction in the fuel devolatilisation around the pyrolysis zone owing to the quantity of heat transferred from the oxidation zone, which as a consequence led to continuous volatilisation, which is also a precursor to the formation of the constituents of the syngas (CO, H_2_, CO_2_, CH_4_, N_2_). As a result of the oxidant’s access into the pyrolysis zone, combustion of the product gases occurred, further leading to a reduction in the quantity of the integral components (CO and H_2_) of the syngas. The syngas composition obtained from gasification actually depends on the nature and composition of the feedstock used since biomass feedstocks vary in composition [62].

**Figure 8:**
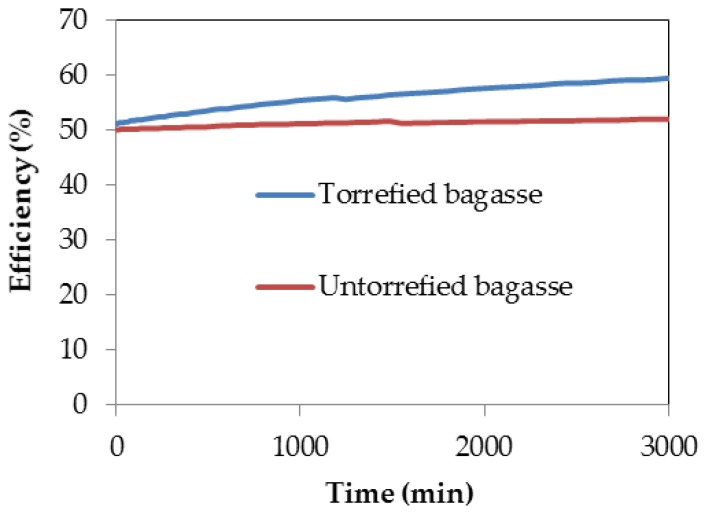
Conversion efficiency obtained after computer simulation of the gasification processes of torrefied and untorrefied SCB.

The conversion efficiency of the gasification processes of both torrefied and untorrefied SCB obtained after computer simulation is presented in Figure 8.

The conversion efficiency of the gasification process of both samples showed that higher efficiency was achieved with torrefied SCB as compared to the efficiency reached by using untorrefied SCB as feedstock. This is evident from Figure 8 and agrees with the fact that the gasification process efficiency of torrefied biomass is always greater than that of untorrefied biomass, as reported by Dorde [32]. This difference in efficiency is significant because the efficiency of torrefied SCB increased with time from about 50% to a maximum percentage of ca. 60%, whereas that of untorrefied SCB began at around 50% efficiency and remained constant even as the gasification time increased. This percentage increase in efficiency between both samples is approximately 14%, which is statistically significant and can be attributed mainly to changes in the properties of SCB after undergoing torrefaction, especially the alteration in chemical composition that resulted in reduced O–C ratio as well as the increased content of C and calorific value as clearly noted in Table 2. With reduced O–C ratio, the gasification characteristics of torrefied biomass materials are improved compared to the untorrefied materials, and efficiency of conversion of a gasification process depends upon the characteristics of the feedstock; low conversion efficiency and poor gas quality including high tar concentration are a consequence of the gasification of low quality biomass [40,62]. The difference in efficiency between the gasification processes of torrefied and untorrefied SCB could also be attributed to the ash catalytic effect owing to the difference in content of catalytic elements in torrefied SCB, although inorganic elemental component analysis may be necessary to establish this fact. High inorganic matter content due to increased content of ash in torrefied biomass may improve gasification efficiency as the rate of gasification also depends on catalytically active constituents of the biomass [62,102].

## 4. Discussion

The solid product obtained from bagasse torrefaction showed improved characteristics when compared with the data obtained for untorrefied bagasse characteristics, especially with regards to carbon content and calorific value, which were raised after torrefaction. A colour change from light brown to a relatively dark colour after torrefaction, as shown in Figure 2a,b, suggests the complete carbonization of torrefied SCB, which corresponded to changes in the physical and chemical properties that further indicate improved characteristics. Following this, a number of reaction products were formed whose compositions and yields relied heavily on certain conditions of torrefaction, with temperature, time and heating rate identified as the most important factors. Among the products obtained from the torrefaction process of sugarcane bagasse (SCB) were solid, liquid and gaseous products in varying proportions. The solid product, identified as the torrefied material, had greater liquid and gaseous yields. The increased yield of torrefied SCB also had an impact on the percentage composition of the syngas obtained during the gasification of the material. According to Xue et al. [62], increased yield of torrefied biomass in terms of weight percentage obtained after torrefaction is construed to mean a higher syngas yield that reflects increased conversion efficiency during gasification. The gaseous products from the torrefaction process (Figure 3b) included CO, CO_2_, H_2_ and CH_4_ in varying proportions and were similar to the composition of the syngas obtained during gasification. The composition and yield of these gases also depended upon several processing factors such as temperature. In addition, the mass and energy yields of the torrefaction process of sugarcane bagasse showed increased yields that also reflected improved properties of the material because of the destruction of the fibrous structure of torrefied SCB, which also had a positive impact on the efficiency achieved during its gasification. Destruction of SCB fibrous structure during torrefaction promoted rising temperatures and higher reactivity during gasification, culminating in increased gasification efficiency for torrefied SCB. This is in agreement with studies undertaken on the impact of torrefaction on properties of *Miscanthus giganteus* relevant to gasification by Xue et al. [62], who concluded that, after torrefaction, the fibrous nature of biomass is destroyed such that when the biomass is used as a feedstock in a gasification process, increasing temperatures and biomass reactivity are instigated with a direct impact on gasification efficiency. Thus, the improved characteristics of torrefied SCB played a role in its gasification performance since a difference in efficiency of about 10% was achieved during computer simulation of the gasification processes of both samples.

Recommended for further studies are the analyses of the liquid products from torrefied SCB and their impact on gasification, the performance of torrefaction of SCB at different temperatures and the effect of varying temperature on mass and energy yields relevant to gasification. The analysis of the mineral matter content of torrefied SCB and their influence on ash composition and on the conversion efficiency of the gasification process conducted under different gasifier operating conditions are also other areas of research worthy of further investigation, as complete feedstock carbon conversion depends on char residual reactivity as well as on the operating conditions of the gasifier [54]. High ash composition was reported for torrefied bagasse, which was attributed to a number of factors that includes the uptake of nutrients during growth of the sugarcane plant. Pith is a tissue in the stem of plants responsible for storing and transporting nutrients through plants. Against this, the gasification characteristics of depithed SCB is also worthy of study so as to establish its effect on the composition of ash as well as determine if depithed SCB is a feedstock suitable for gasification operations, especially using the downdraft gasification system.

Despite global efforts aimed at developing torrefaction technologies, a number of technical and economic issues need to be addressed before full commercialization of this technology [103]. The cost of biomass torrefaction may be offset by higher gasification efficiency of the torrefied biomass; however, not many studies have been undertaken on the techno-economic aspect of torrefaction processes, and even though the gasification of torrefied biomass leads to improved flow properties of the biomass and increased levels of H_2_ and CO in the resulting syngas and enhances overall process efficiencies, comprehensive knowledge and experience are lacking on the possibilities and limitations of the use of torrefied biomass in gasification processes. This is an area where systemic research and development would be extremely useful [104].

## 5. Conclusions

This work investigated the influence of torrefaction on the characteristics and conversion efficiency of the gasification processes of torrefied and untorrefied sugarcane bagasse. The most important analyses (TG, CHNS, GC and SEM) relevant to the use of the two feedstocks studied as gasification feedstocks were undertaken and results indicated that both torrefied and untorrefied SCB are carbonaceous feedstocks well suited for gasification in a downdraft type of system properly developed to accommodate the characteristics of both feedstocks; however, to achieve the desired efficiency, SCB must be taken through a pre-treatment process relevant to gasification to improve its quality and make it more suitable for conversion because increased calorific value, reduced O_2_ content as well as lower O–C and higher H–C ratios including porous structure and larger surface area, as revealed by SEM analysis, are features that led to improved gasification based on efficiency. These features were all observed with torrefied bagasse in this study. The results of the TGA analysis conducted on the samples were also in good agreement with the behaviour of the fuels in downdraft gasification systems. The syngas composition obtained after a computer simulation of the gasification processes of both torrefied and untorrefied SCB exhibited no significant disparity in terms of percentage composition of the individual gases, implying that torrefaction had little or no impact on the volume of syngas produced during gasification of SCB; however, optimum gasification efficiency was achieved with torrefied SCB, making it a more suitable feedstock for gasification than untorrefied SCB.

## Figures and Tables

**Table 1:** Parameters used during computer simulation of the gasification processes of torrefied and untorrefied sugarcane bagasse.

Standard Gasification Conditions [53]		Untorrefied Sugarcane Bagasse (SCB)		Torrefied SCB	
Gasifier operating parameters	Value	Fuel properties	Value	Fuel properties	Value
Throat diameter (cm)	25.5	C (wt %)	44.1	C (wt %)	56.16
Throat angle (°)	30	H (wt %)	5.7	H (wt %)	3.94
Insulation thickness (cm)	17.5	O (wt %)	47.7	O (wt %)	37.27
Thermal conductivity (W·cm^−1^·K)	2.8	N (wt %)	0.20	N (wt %)	1.80
Temperature of input air (K)	300	Fixed carbon (wt %)	18.19	Fixed carbon (wt %)	28.45
Air input (kg·h^−1^)	44.5	Bulk density (g·cm^−3^)	0.178	Bulk density (g·cm^−3^)	1.70
Feed input (kg·h^−1^)	40	Diameter of particle (cm)	14.3	Diameter of particle (cm)	14.3
Heat loss (%)	12.8	Moisture content (%)	1.14	Moisture content (%)	0.87

**Table 2:** Measured key characteristics of torrefied and untorrefied SCB.

PropertiesCaption	Torrefied SCB	Untorrefied SCB
**Proximate Analysis**	(%) ± SD *	(%) ± SD *
Moisture content	0.87 ± 0.01	1.14 ± 0.01
Volatile matter content	30.07 ± 0.01	71.73 ± 0.01
Fixed carbon	28.45 ± 0.01	18.19 ± 0.01
Ash	16.61 ± 0.01	1.32 ± 0.01
**Ultimate Analysis**		
C (%)	56.16 ± 0.22	44.1 ± 0.06
H (%)	3.94 ± 0.05	5.7 ± 0.06
O (%)	37.27 ± 0.01	47.7 ± 0.06
N (%)	1.80 ± 0.01	0.20 ± 0.01
O-C molar ratio	0.50	0.82
H-C molar ratio	0.84	1.55
**Other Properties**		
Calorific value (MJ·kg^−1^)	20.19 ± 0.01	17.86 ± 0.02

* SD represents standard deviation, while SCB depicts sugarcane bagasse. O concentration was calculated by difference in terms of the weight percentages of C, H, and N; while O–C and H–C molar ratios were calculated by converting the mass of each element to moles of each using the atomic masses of the elements and then dividing the moles of one element by the moles of the other. It is worthy to remember that percentages are a ratio multiplied by 100. As such, the mass (in grams) of each element was obtained by assuming a certain overall mass for the material, and 100 g was the overall mass assumed since the composition of the elements are in percentages. For example, the weight percentages of C, H and O presented in the table were converted to moles from their atomic masses and dividing the moles of, for instance, O by that of C to obtain the molar ratio of O–C. The same example can be given for the H–C molar ratio. The values presented are where possible, on a dry matter basis.

## Abbreviations

GHG	Greenhouse gas
FC	Fixed carbon
MC	Moisture content
VMC	Volatile matter content
HHV	Higher heating value, (solid) MJ·kg^−1^ (gas) MJ·Nm^−3^
SCB	Sugarcane bagasse
CV	Calorific value
GC	Gas chromatography
*W_1_*	Weight of biomass prior to torrefaction
*W_2_*	Weight of biomass after torrefaction
*CV_gas_*	Calorific value of gas
*CV_fuel_*	Calorific value of fuel
TGA	Thermogravimetric analysis
DTG	Derivative of thermogravimetry
R_W_	Rate of weight loss
F	Fibre structure
P	Pith
ca.	Approximately
